# P-191. Outcomes of *Clostridioides difficile* Infection in Patients Treated with a Partial versus Complete Course of Fidaxomicin

**DOI:** 10.1093/ofid/ofae631.395

**Published:** 2025-01-29

**Authors:** Caroline Rosario, Ethan P Rausch, Rohit Soman, Kaitlin Beckman, Amanda Lefemine

**Affiliations:** Atrium Health, Charlotte, North Carolina; Atrium Health, Charlotte, North Carolina; Atrium Health, Charlotte, North Carolina; Atrium Health, Charlotte, North Carolina; Atrium Health, Charlotte, North Carolina

## Abstract

**Background:**

Current guidelines recommend fidaxomicin as the preferred treatment for initial and recurrent non-fulminant *Clostridioides difficile* infection (CDI). However, widespread use of fidaxomicin in practice is limited by high cost and inconsistent insurance coverages which may necessitate partial completion of a fidaxomicin course with an alternative antibiotic. Currently, to our knowledge, there is no published literature to describe how this impacts CDI treatment outcomes.

**Figure:**
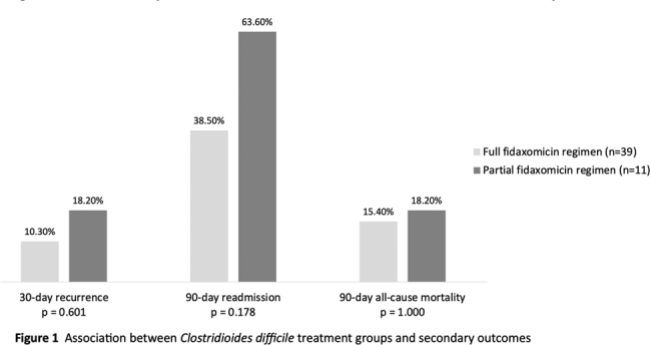

**Methods:**

This was a retrospective, multicenter cohort study of hospitalized patients with CDI treated with fidaxomicin from May 2022 through August 2023. Patients treated with a full course of fidaxomicin (≥20 doses) were compared to those treated with a partial course of fidaxomicin (< 20 doses). Included patients were age ≥18 years, received ≥6 inpatient doses of fidaxomicin, and had a stool sample positive for *C.difficile*. Demographic and clinical data were collected via electronic medical record review. The primary outcome was 90-day CDI recurrence. Secondary outcomes were 30-day CDI recurrence, 90-day hospital readmissions, 90-day all-cause mortality, and hospital length of stay.

**Results:**

The study included 39 patients in the full fidaxomicin group and 11 patients in the partial fidaxomicin group. The population was predominately female (56%) with a mean age of 67.2 years. Among all patients who completed treatment after discharge (62%), a gap in therapy of greater than four days was more frequently observed in the full fidaxomicin group (2/22 vs. 0/9; p=1.000). The partial fidaxomicin group observed a numerically higher rate of 90-day recurrence (18.2% vs. 12.8%; p=0.641). Similar patterns were observed for all secondary outcomes (Figure 1). The mean hospital length of stay was 23.9 in the full fidaxomicin group compared to 9.8 days in the partial fidaxomicin group (p=0.301).

**Conclusion:**

Partial completion of a fidaxomicin course with an alternative antibiotic was associated with a numerically higher rate of CDI recurrence, readmission, and all-cause mortality. Due to small sample size, the study was underpowered to detect statistically significant differences in these findings. Larger comparative effectiveness studies are needed.

**Disclosures:**

**All Authors**: No reported disclosures

